# Association between physical activity levels and healing in people with venous leg ulcers: secondary analysis of prospective cohort data

**DOI:** 10.3389/fmed.2023.1305594

**Published:** 2023-12-21

**Authors:** Yunjing Qiu, Christian R. Osadnik, Natasha K. Brusco, Geoffrey Sussman, Judy Reeves, Leanne Gleghorn, Carolina D. Weller, Victoria Team

**Affiliations:** ^1^School of Nursing and Midwifery, Faculty of Health, University of Technology Sydney, NSW, Australia; ^2^School of Nursing and Midwifery, Faculty of Medicine, Nursing and Health Sciences, Monash University, Clayton, VIC, Australia; ^3^Department of Physiotherapy, School of Primary and Allied Health Care, Faculty of Medicine, Nursing and Health Sciences, Monash University, Frankston, VIC, Australia; ^4^Rehabilitation, Ageing and Independent Living Research Centre, School of Primary and Allied Health Care, Faculty of Medicine, Nursing and Health Sciences, Monash University, Frankston, VIC, Australia; ^5^Austin Health, Melbourne, VIC, Australia; ^6^Department of General Practice Faculty of Medicine, Nursing and Health Science Monash University, Melbourne, VIC, Australia; ^7^Alfred Health, Melbourne, VIC, Australia; ^8^Cabrini Hospital, Malvern, Melbourne, VIC, Australia

**Keywords:** adjuvant treatment, healing, recurrence, physical activity level, varicose ulcer

## Abstract

**Aim:**

To explore the relationship between physical activity levels and wound healing and recurrence in people with venous leg ulcers.

**Methods:**

Questionnaires and medical records were used to collect data, with responses used to group participants into different physical activity groups. The differences in healing and recurrence outcomes of ulcers among different physical activity groups were compared using Chi-square, Kaplan Meier survival analysis, Cox proportional hazards regression analysis, and Kruskal-Wallis test. To measure the strength of the association between physical activity levels and patient outcomes, Spearman’s Rho tests were used. We used descriptive analysis to examine how physical activity levels change over 24 weeks.

**Results:**

Participants were classified into four distinct groups based on physical activity levels reported at baseline and week 12. The survival analysis showed higher physical activity level was associated with a shorter time to healing (log-rank test = 14.78, *df* = 3; *p* = 0.002). The persistently moderate-to-vigorous group had a 7.3-fold increased likelihood of healing compared to the persistently sedentary group. High levels of physical activity were also associated with a better quality of life score at baseline ( ρ = 0.41, *p* < 0.000), week 12 ( ρ = 0.36, *p* < 0.001), and week 24 ( ρ = 0.49, *p* < 0.000). Most participants (48.5%) reported low levels of physical activity, which remained low for the entire study period.

**Conclusion:**

An increased level of physical activity was linked to a shorter healing time and enhanced quality of life. Low levels of physical activity appeared common among people with venous leg ulcers.

## 1 Background

Venous leg ulcer (VLU) is one of the most prevalent chronic ulcers in the lower extremity, and it remains a major challenge in clinical practice (1). Venous ulceration can impact individuals’ quality of life (QoL) due to pain, limited mobility, wound discharge, and offensive odor (2, 3). Compression therapy, such as bandages and hosiery, has been proven to be the best practice for managing people with VLUs as it promotes venous blood return and reduces pressure build-up in the lower limb (4, 5). There is, however, considerable variation in healing times when treating people with compression therapy (6). Even with compression therapy, recurrence rates of ulcers are common, with over half of patients experiencing recurrences within a year after healing (7). This variance in healing time and the high recurrence rate once healed mean that we need to identify adjuvant therapies that may impact healing and recurrence (8).

Physical activity (PA) may be an effective adjunct treatment to compression to improve ulcer healing and recurrence (9). A recent scoping review has found that multicomponent exercises targeting the lower extremity as an adjunct for compression therapy may improve calf muscle pump function, thereby enhancing circulation in the lower extremities and ultimately improving ulcer healing and preventing recurrence (10). There are also multiple barriers to PA and exercising in people with VLUs, including pain, psychological distress, and a lack of clear exercise instructions (11). Nevertheless, questions remain regarding the nature of PA in people with VLUs and how it may change over time. Evidence on how and whether these differences in PA levels affect healing and recurrence is still lacking.

To address the above evidence gap, a study involving repeated measurements on venous ulcer healing status and PA levels over several time points could help inform future work in this area. This study aims to explore the effect of different PA levels healing on healing and recurrence in people with VLUs. The following number of questions were explored in the study:

1. Is there any difference in the healing (e.g., proportion of target ulcers healed, time to healing, and changes in the size of target ulcers) and recurrence outcomes of target ulcers between people with different PA levels?
2. What are the associations between PA level, pain level, and QoL among people with different PA levels?
3. Is there a change in PA level during venous ulceration?

## 2 Methods

### 2.1 Study design

This is a secondary analysis of data from a prospective observational multicenter cohort study. The observational multicenter cohort study was related to the ASPirin in Venous Leg Ulcer (ASPiVLU) randomized control trial that examined the effect of aspirin as an adjunct treatment to compression on venous ulcer healing within 12 weeks period (12). The ASPiVLU observational cohort study data was collected from five outpatient chronic wound clinics in Queensland, New South Wales, Victoria, and Tasmania between 2017–2018. Ethics approval was granted by the Human Ethics Committees of Alfred Health and Monash University.

### 2.2 Participants

This study included people who (1) were aged 18 and over; (2) have experienced at least one active VLU for at least 6 weeks or had a prior history of venous ulceration. This study excluded people who (1) were unable to understand English; (2) were unable to provide consent. A total of 210 participants were included in this cohort study.

### 2.3 Data sources

Self-reported questionnaires were used to collect data from participants. Questionnaires were distributed to study participants for completion in person during the baseline visit. Subsequent questionnaires were sent by mail to study participants at 12 and 24 weeks following the initial screening visit. Questionnaires collected information on the healing and recurrence status of their target VLUs, pain scores, QoL ratings, and PA levels. Medical records were accessed at baseline to collect demographic information (e.g., age, gender, and medical history), and at week 24 to extract routine care information and verify the status of participant-reported VLU healing and/or recurrence (as reported via direct clinician observation) during the study period. The cohort data did not include the details of the standard treatment, and no data was collected on the type of compression and VLU dressings.

### 2.4 Measures

#### 2.4.1 PA levels among people with VLUs

The Rapid Assessment of PA instrument was used to assess the level of PA (13). The Rapid Assessment of PA instrument was created with the purpose of evaluating the routine PA engagement of the elderly, encompassing aspects such as its type, quantity, and intensity (13). Participants were asked to choose one out of the seven statements indicating ‘sedentary’, ‘light PA level’, or ‘moderate-to-vigorous PA level [MVPA]’ at baseline, week 12, and week 24 (13).

#### 2.4.2 Proportion of target ulcer healed

The target ulcer healing rate was measured at baseline, week 12, and week 24. Closed-ended question (yes or no) was asked on the questionnaire to determine the healing status of participants’ target ulcers. The term ‘healing’ refers to epithelization without scabs (14). The healing status of VLU was determined by qualified wound care specialists at regular clinic visits.

#### 2.4.3 Time to healing of target ulcer

Time to healing of participants’ target ulcers was measured at week 12 and week 24. The time to healing of each participant was calculated by the date his/her target ulcer healed minus the date of his/her target ulcer was diagnosed.

#### 2.4.4 Changes in target ulcer size

Changes in target ulcer size were measured at baseline, week 12, and week 24. This information allowed for the determination of ulcer area reduction in participants who did not heal completely. Participants were asked if their target ulcers had ‘decreased’, ‘increased’, or ‘same size’ at each time point. Medical records were also used to obtain the accurate size of target ulcers at week 24.

#### 2.4.5 Recurrence of target ulcer

The recurrence of the target ulcer was measured at weeks 12 and 24 in participants whose ulcers had healed. Recurrence was defined as a new VLU occurring on the same leg that was affected previously (15). Participants were asked in the questionnaires if they had ulcer recurrence at each time point by responding ‘yes’ or ‘no’. As study eligibility criteria allowed for the inclusion of people with both active and prior ulcers at the time of recruitment, ulcer recurrence definition pertained to the event of a new ulcer being detected. For participants recruited with active ulcers, this indicates ulcer healing and subsequent recurrence during the study period, while for those recruited in a healed status, this indicates the onset of a new ulcer.

#### 2.4.6 Health-related quality of life

Health-related QoL was measured at baseline, week 12, and week 24 by using EQ-5D-5L, a generic tool for measuring QoL that has been shown to be valid and reliable for measuring QoL scores in people with VLUs (16, 17). EQ-5D-5L assesses patients’ QoL from five aspects, including mobility, self-care, usual activities, pain/discomfort, and anxiety/depression (18). Each aspect has five levels (no problems, slight problems, moderate problems, severe problems and extreme problems), and participants were asked to indicate their health status at each aspect by selecting the most appropriate level (18).

For this study, utility score derived from the raw scores using the United Kingdom value set and scoring algorithm, since Australian value set and scoring algorithms was not available (19).

#### 2.4.7 Wound pain score

Pain level was measured at baseline, week 12 and week 24. Participants were asked to rate their pain on a numerical rating scale of 0–10, with 0 indicating this was the least painful, and 10 indicating the worst pain.

### 2.5 Data analysis

**Figure 1:**
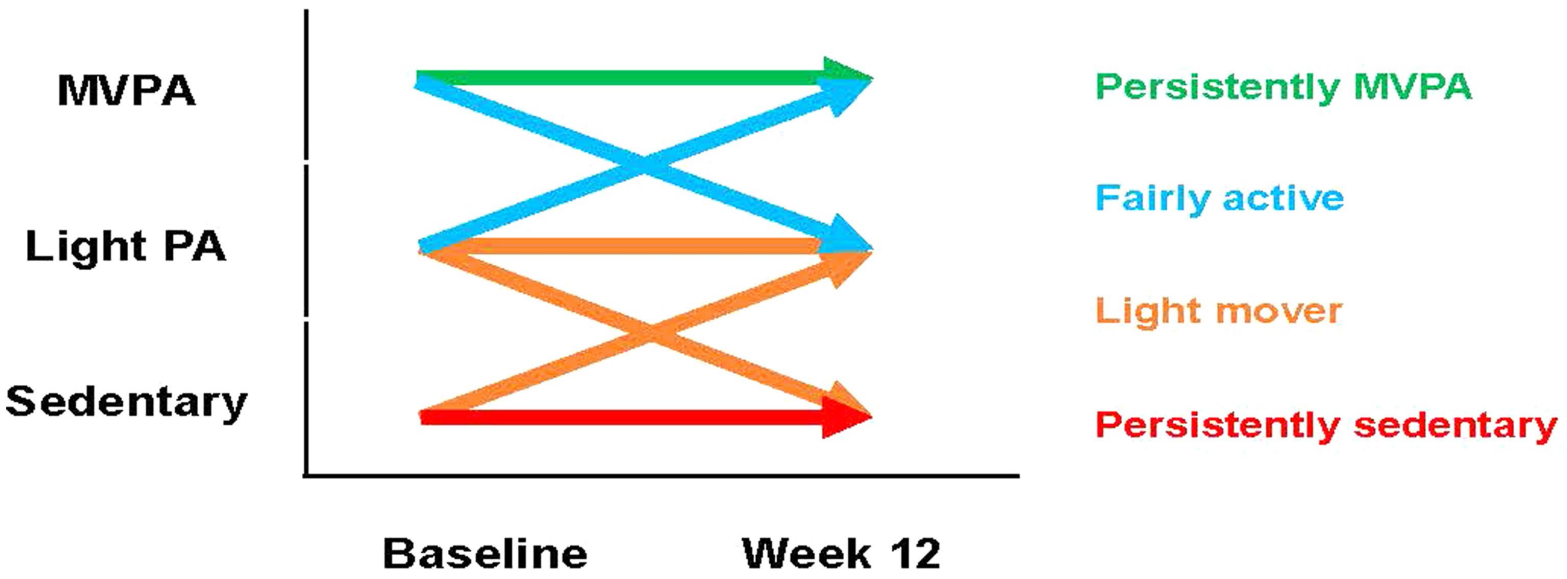
Overview of how each PA is classified. MVPA, moderate-to-vigorous physical activity; PA, physical activity.

Changes in self-reported PA levels throughout the 24 weeks study period were defined according to Rapid Assessment of PA responses (grouped into three logical groups - ‘sedentary’, ‘light PA’, ‘MVPA’) and represented visually via Sankey diagram for those who had complete data available across all three study time points (baseline, week 12, week 24). All other analyses relating to the impact of PA on study outcomes involved re-grouping individuals according to their status over the first 12 weeks of the study. This process to derive four key PA groups is summarized in Figure 1.

Descriptive analyses were undertaken to characterize the study sample for key demographic characteristics by different PA groups. Chi-squared test was performed to determine the differences of the following categorical variable among four PA level groups at both week 12 and week 24: the proportion of target ulcer healed/not healed, changes in target ulcer size, and target ulcer recurrences. Kaplan Meier survival analysis, and cox regression proportional hazards model were performed to determine whether there was a difference in time to healing between different PA groups within the 24 weeks study period. The Kruskal-Wallis test was used to compare the changes in target ulcer size among PA level groups. The Spearman’s Rho test was used to measure the strength of association between PA levels and the following variables: quality of life, and pain score. We used Stata version 16 (16.1, StataCorp LLC, College Station, TX) for all analyses with statistical significance set at *p* < 0.05.

## 3 Results

### 3.1 Characteristics of participants

A total of 210 people with VLUs were recruited. Among these participants, 104 (49.5%) people completed self-reported questionnaires, reported their PA levels, and returned the questionnaires at baseline, week 12, and week 24. The average age of all participants was 74 (SD 11) years old, with an average target ulcer duration of 5 months [interquartile range (IQR:3–14) (Table 1)]. There was equal representation between male (52.9%) and female (47.1%) participants in this cohort. The most common comorbidities among these participants were cardiovascular diseases (57.7%) and hypertension (67.3%). Over 80% of participants reported that they were not working. The majority of participants stated that they experience minor (29.1%) to moderate (25.5%) mobility issues. In comparison with participants in the other three groups, participants in the persistently MVPA group had higher mean QoL scores [EQ-5D-5L utility score: 0.8 (IQR:0.7–0.8); EQ-VAS score 80 (IQR:60–90) versus EQ-5D-5L utility score ‘persistently sedentary’:0.4 (IQR:0.1–0.6); ‘light mover’: 0.6 (IQR:0.5–0.8); ‘fairly active’:0.7 (IQR:0.7–0.8); EQ-VAS score ‘persistently sedentary’:52.5 (IQR:35–65); ‘light mover’:70 (IQR:50–85); ‘fairly active’:72.5 (IQR:67.5–85)]. Similarly, participants in the persistently MVPA group had fewer VLUs in their lifetime than the others.

**Table 1:** Summary of socio-demographic and health-related characteristics by levels of physical activity.

Baseline characteristics	Total (*n* = 104)	Persistently sedentary (*n* = 12)	Light mover (*n* = 55)	Fairly active (*n* = 24)	Persistently MVPA (*n* = 13)
Age (year) ^*^	74 (11)	73 (11)	76 (12)	72 (12)	69 (7)
Gender [*n* (%)] ^&^					
Male	55 (52.9)	9 (75.0)	23 (41.8)	17 (70.8)	6 (46.2)
Female	49 (47.1)	3 (25.0)	32 (58.2)	7 (29.2)	7 (53.9)
Comorbidities [*n* (%)]^&^					
Diabetes	32 (30.8)	7 (58.3)	15 (27.3)	9 (37.5)	1 (7.7)
Cardiovascular diseases	60 (57.7)	11 (91.7)	35 (63.6)	12 (50.0)	2 (15.4)
Hypertension	70 (67.3)	9 (75.0)	39 (70.9)	14 (58.3)	8 (61.5)
Hip osteoarthritis	12 (11.5)	2 (16.7)	6 (10.9)	4 (16.7)	0 (0.0)
Knee osteoarthritis	35 (33.7)	5 (41.7)	19 (34.6)	6 (25.0)	5 (38.5)
Rheumatoid arthritis	6 (5.8)	1 (8.3)	4 (7.3)	0 (0.0)	1 (7.7)
Deep vein thrombosis	21 (20.2)	2 (16.7)	12 (21.8)	7 (29.2)	0 (0.0)
Smoking status [*n* (%)]^&^					
Never smoke	54 (51.9)	5 (41.7)	31 (56.4)	12 (50.0)	6 (47.2)
Past smoker	42 (40.4)	5 (41.7)	19 (34.6)	11 (45.8)	7 (53.9)
Current smoker	8 (7.7)	2 (16.7)	5 (9.1)	1 (4.2)	0 (0.0)
Employment [*n* (%)]^&^					
Full-time	13 (12.5)	2 (16.7)	6 (10.9)	4 (16.7)	1 (7.7)
Part-time	6 (5.8)	0 (0.0)	1 (1.8)	2 (8.3)	3 (23.1)
Working from home	2 (1.9)	0 (0.0)	0 (0.0)	1 (4.2)	1 (7.7)
Casual	3 (2.9)	0 (0.0)	2 (3.6)	1 (4.2)	0 (0.0)
Not working	80 (76.9)	10 (83.3)	46 (83.6)	16 (66.7)	8 (61.5)
Mobility [*n* (%)]^&^					
No problems with walking around	27 (26.5)	1 (8.3)	13 (23.6)	5 (22.7)	8 (61.5)
Slight problems with walking around	30 (29.4)	4 (33.3)	14 (25.5)	9 (40.9)	3 (23.1)
Moderate problems with walking around	26 (25.5)	2 (16.7)	16 (29.1)	6 (27.3)	2 (15.4)
Severe problems with walking around	17 (16.7)	4 (33.3)	11 (20.0)	2 (9.1)	0 (0.0)
Unable to walk around	2 (2.0)	1 (8.3)	1 (1.8)	0 (0.0)	0 (0.0)
Ankle range of motion [*n* (%)]^&^					
Full range	61 (77.2)	5 (62.5)	31 (79.5)	14 (73.7)	11 (84.6)
Reduced range	18 (22.8)	3 (37.5)	8 (20.5)	5 (26.3)	2 (15.4)
Duration of target ulcer (months) ^#^	5 [3–14]	4 [3–8.5]	6 [3–16]	4.5 [1.5–10]	3 [2–7]
Description of target ulcer [*n* (%)]^&^					
Over bone	42 (41.6)	6 (50.0)	23 (43.4)	10 (43.5)	3 (23.1)
Soft tissue	57 (56.4)	6 (50.0)	28 (52.8)	13 (56.5)	10 (76.9)
Circumferential	2 (2.0)	0 (0.0)	2 (3.8)	0 (0.0)	0 (0.0)
The number of venous leg ulcers have patients had in their lifetime [*n* (%)]^&^					
1	35 (34.0)	3 (25.0)	18 (33.3)	12 (50.0)	2 (15.4)
2–4	37 (35.9)	4 (33.3)	19 (35.2)	7 (29.2)	7 (53.9)
5–7	16 (15.5)	2 (16.7)	9 (16.7)	4 (16.7)	1 (7.7)
≥8	15 (14.5)	3 (25)	8 (14.8)	1 (4.2)	3 (23.1)
Current compression usage [*n* (%)]^&^					
Yes	97 (94.2)	12 (100.0)	50 (90.9)	23 (100.0)	12 (92.3)
No	6 (5.8)	0 (0.0)	5 (9.1)	0 (0.0)	1 (7.7)
Pain score ^#^	3 [0–6]	5.5 [1.5–8.5]	4 [0–6]	2 [0–5]	3 [1–4]
EQ-5D-5L utility score ^#^	0.7 [0.6–0.7]	0.4 [0.1–0.6]	0.6 [0.5–0.8]	0.7 [0.7–0.8]	0.8 [0.7–0.8]
EQ-VAS score ^#^	70 [50–81]	52.5 [35–65]	70 [50–85]	72.5 [67.5–85]	80 [60–90]
Have patients received education on venous leg ulcer? [*n* (%)]^&^					
Yes	88 (87.1)	7 (58.3)	51 (92.7)	20 (87.0)	10 (90.9)
No	13 (12.9)	5 (41.7)	4 (7.3)	3 (13.0)	1 (9.1)

^*^Data are mean (SD). ^#^Data are presented as Median [IQR]; MVPA, moderate-to-vigorous physical activity. ^&^The percentage was calculated based on the available number of cases.

### 3.2 Changes in PA level

At baseline, 24.7% of people reported being sedentary, 48.5% reported being lightly active, and 26.8% reported being moderate to vigorously active (Appendix Figure A1). Approximately one-fourth of the study participants reported being sedentary throughout the entire study period. There was a noticeable rise in people self-reporting moderate PA after week 12 (22.7% week 12, 29.9% week 24) (Appendix Figure A1).

### 3.3 Healing status of target ulcers

**Figure 2:**
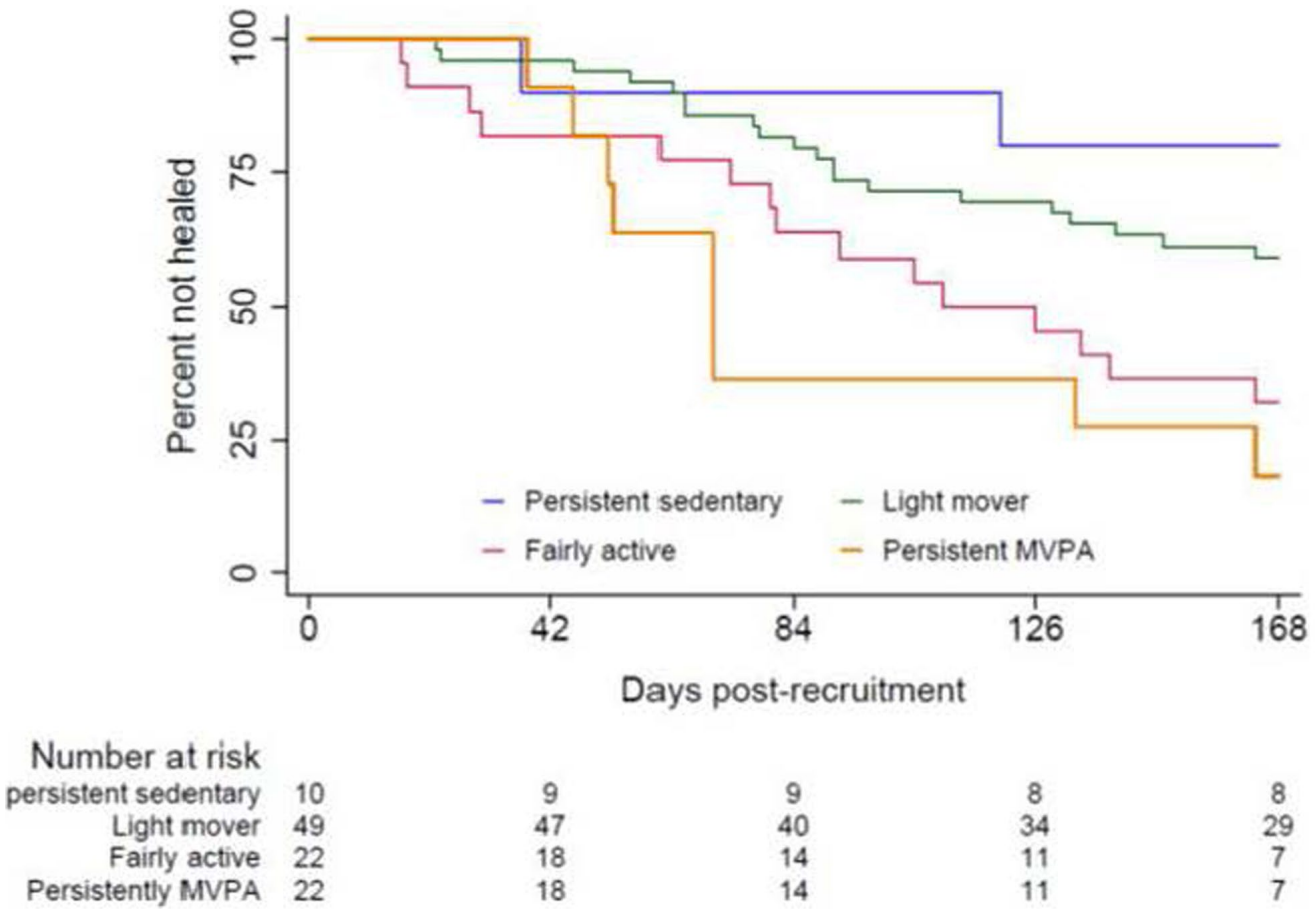
Kaplan-Meier analysis of the time to healing among four PA groups; *p* = 0.002 between-groups. MVPA, moderate-to-vigorous physical activity; For an explanation of each group categorization, please refer to the definition of each PA group provided in Figure 1.

Over half of the participants in the persistently MVPA group reported ulcers healed by the end of week 12 (Table 2). At week 24, the difference in the proportion of participants who had their target ulcers healed became more apparent. Specifically, 69.2% of participants in the persistently MVPA group had their target ulcers healed, compared to 25.0% of participants in the persistently sedentary group, 38.1% of participants in the light mover group, and 62.5% of participants in the fairly active group (Table 2). The Kaplan–Meier curve indicated that participants who have higher PA levels healed faster than the participants have low PA levels (log-rank test = 14.78, *df* = 3; *p* = 0.002) (Figure 2), with differentiation between groups apparent after approximately 53 days (Figure 2). By the end of week 24 (168 days), approximately 18.2% of participants in the persistently MVPA group had active ulcers, while over 75% of participants in the persistently sedentary group had active ulcers. The Cox regression model revealed that participants in the persistently MVPA group, fairly active group, and light mover group had a 7.3, 4.8, and 2.2-fold increased likelihood of healing, respectively, compared to the persistently sedentary group (Table 3), however, findings were not statistically significant for the light mover group.

**Table 2:** Healing status of target venous ulcer among four PA groups.

	Week 12				Week 24			
	Persistently sedentary (*n* = 12)	Light mover (*n* = 55)	Fairly active (*n* = 24)	Persistently MVPA (*n* = 13)	Persistently sedentary (*n* = 12)	Light mover (*n* = 55)	Fairly active (*n* = 24)	Persistently MVPA (*n* = 13)
Healed, *n* (%) ^a^	2 (16.7)	11 (20.0)	9 (37.5)	7 (53.9)	3 (25.0)	21 (38.1)	15 (62.5)	9 (69.2)
Changes in ulcer size (non-healed), *n* (%) ^a^								
Decreased	10 (83.3)	28 (50.9)	11 (45.8)	6 (46.1)	7 (58.3)	14 (25.4)	5 (20.8)	1 (7.6)
Same size	0 (0.0)	8 (14.5)	2 (8.3)	0 (0.0)	0 (0.0)	8 (14.5)	1 (4.1)	1 (7.6)
Increased	0 (0.0)	7 (12.7)	1 (4.2)	0 (0.0)	1 (8.3)	7 (12.7)	1 (4.1)	0 (0.0)

MVPA, moderate-to-vigorous physical activity; ^a^Percentages were calculated relative to available data only (i.e., small number of missing values due to participant non-response).

**Table 3:** Relationship between different PA levels and healing status of target venous leg ulcers.

	Hazard ratio	Z – Stat	95% CI	*p* value*
Light mover	2.28	1.11	0.53–9.77	0.27
Fairly active	4.79	2.08	1.09–20.98	0.04
Persistently MVPA	7.32	2.54	1.57–34.06	0.01

*Cox proportional hazards regression analysis; MVPA – moderate-to-vigorous physical activity.

### 3.4 Recurrence of target ulcer

Twenty-four participants experienced at least one ulcer recurrence during the study period. Compared to the other physical activity groups, the persistently MVPA group had the lowest recurrence rate at week 12, with only 7.6% of participants experiencing ulcer recurrence. By week 24, the recurrence rate in the persistently MVPA group had decreased to 0%. The 0% recurrence rate observed in the persistently MVPA group indicated that study participants in this group who experienced recurring ulcers at week 12 had achieved healing by week 24. There were no statistically significant differences between the groups (Table 4).

**Table 4:** Recurrence rate of target venous ulcer among four PA groups.

	Week 12				Week 24			
	Persistently sedentary (*n* = 12)	Light mover (*n* = 55)	Fairly active (*n* = 24)	Persistently MVPA (*n* = 13)	Persistently sedentary (*n* = 12)	Light mover (*n* = 55)	Fairly active (*n* = 24)	Persistently MVPA (*n* = 13)
Recurrence of target ulcer, *n* (%)^a^	3 (25.0)	7 (12.7)	3 (12.5)	1 (7.6)	1 (8.3)	6 (10.9)	3 (12.5)	0 (0.0)

MVPA, moderate-to-vigorous physical activity. ^a^The recurrence data included study participants whose venous leg ulcers had presented for at least six weeks or who had healed ulcers at the time of recruitment (please refer to the participants’ inclusion criteria).

### 3.5 Pain score

PA levels demonstrated a weak negative correlation with pain scores at baseline [ ρ (Spearman’s rho) = −0.13, *N* = 151, *p* = 0.10] and a weak positive correlation at week 12 ( ρ = 0.10, *N* = 80, *p* = 0.36) and week 24 ( ρ = −0.09, *N* = 60, *p* = 0.50) (Appendix B). A similar pattern was also seen in the association between changes in PA levels and changes in pain score from baseline to week 12, and from week 12 to week 24.

### 3.6 Quality of life

There was a strong, positive association between PA levels and QoL score at baseline ( ρ = 0.41, *N* = 146, *p* < 0.000), week 12 ( ρ = 0.36, *N* = 108, *p* < 0.001), and week 24 ( ρ = 0.49, *N* = 118, *p* < 0.000) (Appendix C). No association was found between changes in PA levels and changes in QoL score at any of the the time points in this study (Appendix C).

## 4 Discussion

This study is the first to examine the impact of different levels of PA on the healing and recurrence outcomes of VLUs. Results suggest increased levels of PA may have positive effects on both the time to healing and the proportion of people who heal, and that healing is likely influenced by not only the amount of PA but also the intensity at which it is accrued. These findings add to a growing body of indirect evidence and provide important insights to guide future studies seeking to develop effective PA interventions to improve clinical practice.

It is interesting to speculate the possible mechanism to explain the observed benefits that PA levels had on wound healing in the present study. Previous research found that PA can improve the healing outcomes of VLUs because it stimulates calf muscle pump function. The efficiency of calf muscle pump function was closely related to the healing outcomes of VLUs (20). It is plausible that people with higher PA levels in the present study may have had better calf muscle pump function than those with low PA levels however this was not directly measured. Furthermore, our study did not fully explore the interaction between PA/exercise characteristics (i.e., intensity, duration, dosage) and ulcer healing. A number of questions remain unanswered, such as what the minimum volume/ intensity/duration of PA/exercise is needed to derive optimal benefits on wound healing. Future clinical trials in this field will be essential to offer more thorough and detailed information.

Our data suggested higher levels of PA were positively correlated with better QoL. This is consistent with prior research examining factors that influence PA levels in people with VLUs, where a strong positive correlation between PA levels and overall QoL in this patient population was also identified (10, 21). Similarly, past clinical trials found that participants who undertook an exercise intervention had higher QoL scores than those who received standard compression treatment (22, 23). Our study design was not appropriate to confirm cause and effect, however, it is interesting to speculate the extent to which higher PA levels may have contributed to better quality of life scores. People with VLUs commonly experience pain, emotional distress, and negative perceptions of themselves, all of which can negatively affect their quality of life (24, 25). If PA can be shown to alter one’s perception of distress or oneself, it could prove a novel approach to improving this outcome that is of high importance to patients and healthcare providers alike.

Self-reported PA levels of many participants in the present study were concerningly low and stagnant throughout the follow-up period (Appendix Figure A1), with most study participants failing to meet recommended World Health Organization (WHO) PA targets (26). This finding is comparable with the other studies assessing PA levels among people with VLUs (20). Past research also highlighted the importance of enhancing mobility and found that reduced mobility can lead to a decrease in CMPF levels, potentially resulting in venous outflow issues and microcirculation dysfunction in the lower extremities, ultimately leading to VLU formation (27). Key barriers affecting PA participation in this patient group include conflicting/unclear advice from clinicians, discomfort related to the ulcer site, and a fear of falling (11, 28). Given the multifaceted challenges faced by people with VLUs that hinder their PA engagement, a recent study has explored strategies to improve their mobility (29). The research suggested that establishing realistic goals tailored to the challenges encountered by VLU patients and offering flexible exercise programs could enhance people with VLUs participating in PA (29).

## 5 Limitations

There are some limitations in the present study. Firstly, due to the nature of the cohort study, this study cannot prove a causal relationship between PA levels and ulcer healing. Study findings, however, point to a future direction for further research. Secondly, in this study, the time to healing outcomes were measured at certain fixed time points, not daily monitoring. Thirdly, self-reported questionnaires were used to obtain participants’ PA levels, so there could be a response bias in their responses, as most people generally overestimate how active they really are. This study had a small number of participants, consequently affecting the statistical power, and its ability to draw strong conclusions. This study, however, represents an important first step as an exploratory study, demonstrating the association between the level of PA and VLU healing. Findings of this study hold significant value for future research and clinical practice, particularly when designing exercise programs for VLU patients. Lastly, as this was an observational study involving routine care, it was not possible to control for the potentially confounding influence of various therapeutics (e.g., aspirin, compression therapies, and various wound dressings) that were administered taking into account the participants’ individual needs.

## 6 Conclusion

This is the first cohort study to examine the role of PA levels in VLU healing and recurrence outcomes, as well as changes in PA levels during venous ulceration in this patient group. The results of this study indicated that a higher level of PA would greatly improve the healing rate of venous ulcers and shorten their healing period. The effect of different PA levels on ulcer recurrence remained unclear. Our study found that participants with a high level of PA reported better overall QoL scores than those with a low level of PA. Most study participants reported light PA levels, and that level remained stable during the ulceration period. Findings of this study suggest there is a need to promote PA in this population. Further research is recommended to explore clinicians’ perspectives on how and what strategies can be used to support this cohort to engage in PA. Our study findings will be useful in designing a feasible PA intervention for this patient population.

## Data availability statement

The original contributions presented in the study are included in the article/Supplementary material, further inquiries can be directed to the corresponding author.

## Ethics statement

The studies involving humans were approved by Alfred Hospital Ethics Committee and Monash University Ethics Committee. The studies were conducted in accordance with the local legislation and institutional requirements. The participants provided their written informed consent to participate in this study.

## Author contributions

YQ: Conceptualization, Data curation, Formal analysis, Funding acquisition, Investigation, Methodology, Project administration, Resources, Software, Validation, Visualization, Writing – original draft, Writing – review & editing. CO: Conceptualization, Data curation, Formal analysis, Investigation, Software, Supervision, Validation, Visualization, Writing – review & editing. NB: Data curation, Formal analysis, Investigation, Validation, Visualization, Writing – review & editing. GS: Writing – review & editing. JR: Writing – review & editing. LG: Writing – review & editing. CW: Conceptualization, Funding acquisition, Methodology, Resources, Supervision, Writing – review & editing. VT: Conceptualization, Investigation, Methodology, Resources, Supervision, Validation, Writing – review & editing.

## References

[1] Xie T Ye J Rerkasem K Mani R. The venous ulcer continues to be a clinical challenge: an update. Burns Trauma. (2018) 6:18. doi: 10.1186/s41038-018-0119-y, PMID: 29942813 PMC6003071

[2] Barnsbee L Cheng Q Tulleners R Lee X Brain D Pacella R. Measuring costs and quality of life for venous leg ulcers. Int Wound J. (2019) 16:112–21. doi: 10.1111/iwj.13000, PMID: 30289621 PMC7948561

[3] Liu S Team V Qiu Y Weller CD. Investigating quality of life instrument measurement properties for adults with active venous leg ulcers: a systematic review. Wound Repair Regen. (2022) 29:109. doi: 10.33235/wpr.29.2.104-109 PMC9545457 35639021

[4] O'Meara S Cullum N Nelson EA Dumville JC. Compression for venous leg ulcers. Cochrane Database Syst Rev. (2012) 11:182. doi: 10.1002/14651858.CD010182 PMC7068175 23152202

[5] Weller CD. Compression improves healing of venous leg ulcers compared with no compression, with differences between different compression systems. Evid Based Nurs. (2013) 16:94–4. doi: 10.1136/eb-2012-101201, PMID: 23435371

[6] Karanikolic V Karanikolic A Petrovic D Stanojevic M. Prognostic factors related to delayed healing of venous leg ulcer treated with compression therapy. Dermatol Sin. (2015) 33:206–9. doi: 10.1016/j.dsi.2015.04.005

[7] Finlayson KJ Parker CN Miller C Gibb M Kapp S Ogrin R. Predicting the likelihood of venous leg ulcer recurrence: the diagnostic accuracy of a newly developed risk assessment tool. Int Wound J. (2018) 15:686–94. doi: 10.1111/iwj.12911, PMID: 29536629 PMC7949606

[8] Team V Chandler PG Weller CD. Adjuvant therapies in venous leg ulcer management: a scoping review. Wound Repair Regenerat. (2019) 27:562–90. doi: 10.1111/wrr.12724, PMID: 31025794

[9] Lim CS Baruah M Bahia SS. Diagnosis and management of venous leg ulcers. British Med J. (2018) 362:k3115–5. doi: 10.1136/bmj.k3115 30108047

[10] Qiu Y Osadnik CR Team V Weller CD. Effects of physical activity as an adjunct treatment on healing outcomes and recurrence of venous leg ulcers: a scoping review. Wound Repair Regen. (2022) 30:172–85. doi: 10.1111/wrr.12995 35142412 PMC9303258

[11] Qiu Y Team V Osadnik CR Weller CD. Barriers and enablers to physical activity in people with venous leg ulcers: a systematic review of qualitative studies. Int J Nurs Stud. (2022) 135:104329. doi: 10.1016/j.ijnurstu.2022.104329 35986960

[12] Weller CD Barker A Darby I Haines T Underwood M Ward S. Aspirin in venous leg ulcer study (ASPiVLU): study protocol for a randomised controlled trial. Trials. (2016) 17:192. doi: 10.1186/s13063-016-1314-4, PMID: 27068695 PMC4827178

[13] Topolski TD LoGerfo J Patrick DL Williams B Walwick J Patrick MMB. Peer reviewed: the rapid assessment of physical activity (RAPA) among older adults. Prev Chronic Dis. (2006) 3:A118. PMID: 16978493 PMC1779282

[14] Jull A Parag V Walker N Maddison R Kerse N Johns T. The prepare pilot RCT of home-based progressive resistance exercises for venous leg ulcers. J Wound Care. (2009) 18:497–503. doi: 10.12968/jowc.2009.18.12.45606 20081574

[15] Parker CN Finlayson KJ Edwards HE. Predicting the likelihood of delayed venous leg ulcer healing and recurrence: development and reliability testing of risk assessment tools. Ostomy Wound Manage. (2017) 63:16–33. PMID: 29091035

[16] Ashby RL Gabe R Ali S Adderley U Bland JM Cullum NA. Clinical and cost-effectiveness of compression hosiery versus compression bandages in treatment of venous leg ulcers (venous leg ulcer study IV, VenUS IV): a randomised controlled trial. Lancet. (2014) 383:871–9. doi: 10.1016/S0140-6736(13)62368-5 24315520

[17] Cheng Q Kularatna S Lee XJ Graves N Pacella RE. Comparison of EQ-5D-5L and SPVU-5D for measuring quality of life in patients with venous leg ulcers in an Australian setting. Qual Life Res Int J Qual Life Asp Treat Care Rehab. (2019) 28:1903–11. doi: 10.1007/s11136-019-02128-6, PMID: 30778889

[18] Herdman M Gudex C Lloyd A Janssen MF Kind P Parkin D. Development and preliminary testing of the new five-level version of EQ-5D (EQ-5D-5L). Qual Life Res. (2011) 20:1727–36. doi: 10.1007/s11136-011-9903-x, PMID: 21479777 PMC3220807

[19] Devlin NJ Shah KK Feng Y Mulhern B van Hout B. Valuing health-related quality of life: an EQ-5D-5L value set for England. Health Econ. (2017) 27:7–22. doi: 10.1002/hec.356, PMID: 28833869 PMC6680214

[20] O'Brien JA Edwards HE Finlayson KJ Kerr G. Understanding the relationships between the calf muscle pump, ankle range of motion and healing for adults with venous leg ulcers: a review of the literature. Wound Pract Res. (2012) 20:80–5.

[21] Smith D Team V Barber G O'Brien J Wynter K McGinnes R. Factors associated with physical activity levels in people with venous leg ulcers: a multicentre, prospective, cohort study. Int Wound J. (2018) 15:291–6. doi: 10.1111/iwj.12868, PMID: 29266735 PMC7950050

[22] Klonizakis M Tew GA Gumber A Crank H King B Middleton G. Supervised exercise training as an adjunct therapy for venous leg ulcers: a randomized controlled feasibility trial. Br J Dermatol. (2018) 178:1072–82. doi: 10.1111/bjd.16089, PMID: 29077990 PMC6001633

[23] O'Brien J Finlayson K Kerr G Edwards H. Evaluating the effectiveness of a self-management exercise intervention on wound healing, functional ability and health-related quality of life outcomes in adults with venous leg ulcers: a randomised controlled trial. Int Wound J. (2017) 14:130–7. doi: 10.1111/iwj.12571, PMID: 26817648 PMC7949716

[24] Gonzalez de la Torre H Quintana-Lorenzo ML Perdomo-Pérez E Verdú J. Correlation between health-related quality of life and venous leg ulcer's severity and characteristics: a cross-sectional study. Int Wound J. (2017) 14:360–8. doi: 10.1111/iwj.12610, PMID: 27112627 PMC7949864

[25] Vishwanath V. Quality of life: venous leg ulcers. Indian Dermatol Online J. (2014) 5:397–9. doi: 10.4103/2229-5178.137828, PMID: 25165684 PMC4144252

[26] World Health Organization. (2020). WHO guidelines on physical activity and sedentary behavior: at a glance [internet]. Available at: https://apps.who.int/iris/bitstream/handle/10665/337001/9789240014886-eng.pdf (Accessed June 18, 2022)

[27] Meulendijks AM Franssen WMA Schoonhoven L Neumann HAM. A scoping review on chronic venous disease and the development of a venous leg ulcer: the role of obesity and mobility. J Tissue Viability. (2020) 29:190–6. doi: 10.1016/j.jtv.2019.10.002, PMID: 31668667

[28] Kesterton S Crank HJ Tew GA Michaels J Gumber A McIntosh E. Participant experiences in a feasibility trial of supervised exercise training in adults with venous leg ulcers: a qualitative study. Int Wound J. (2019) 16:1559–69. doi: 10.1111/iwj.13252, PMID: 31606948 PMC7949412

[29] Qiu Y Osadnik CR Team V. Planning exercise interventions as an adjunct treatment in managing venous leg ulcers: a qualitative study of nurses' perspectives. J Tissue Viability. (2023) 32:279–85. doi: 10.1016/j.jtv.2023.03.004, PMID: 37032305

