# Supplementary material for: Association between physical activity levels and healing in people with venous leg ulcers: secondary analysis of prospective cohort data
Source: Front Med (Lausanne). 2023 Dec 21;10:1305594. doi: 10.3389/fmed.2023.1305594 (PMC10768026; doi:10.3389/fmed.2023.1305594)
Supplement: Supplementary file 1 [file Table_1.DOCX]

**Appendix A.**

Baseline Week 12 Week 24

(n= 97) (n=97) (n=97)


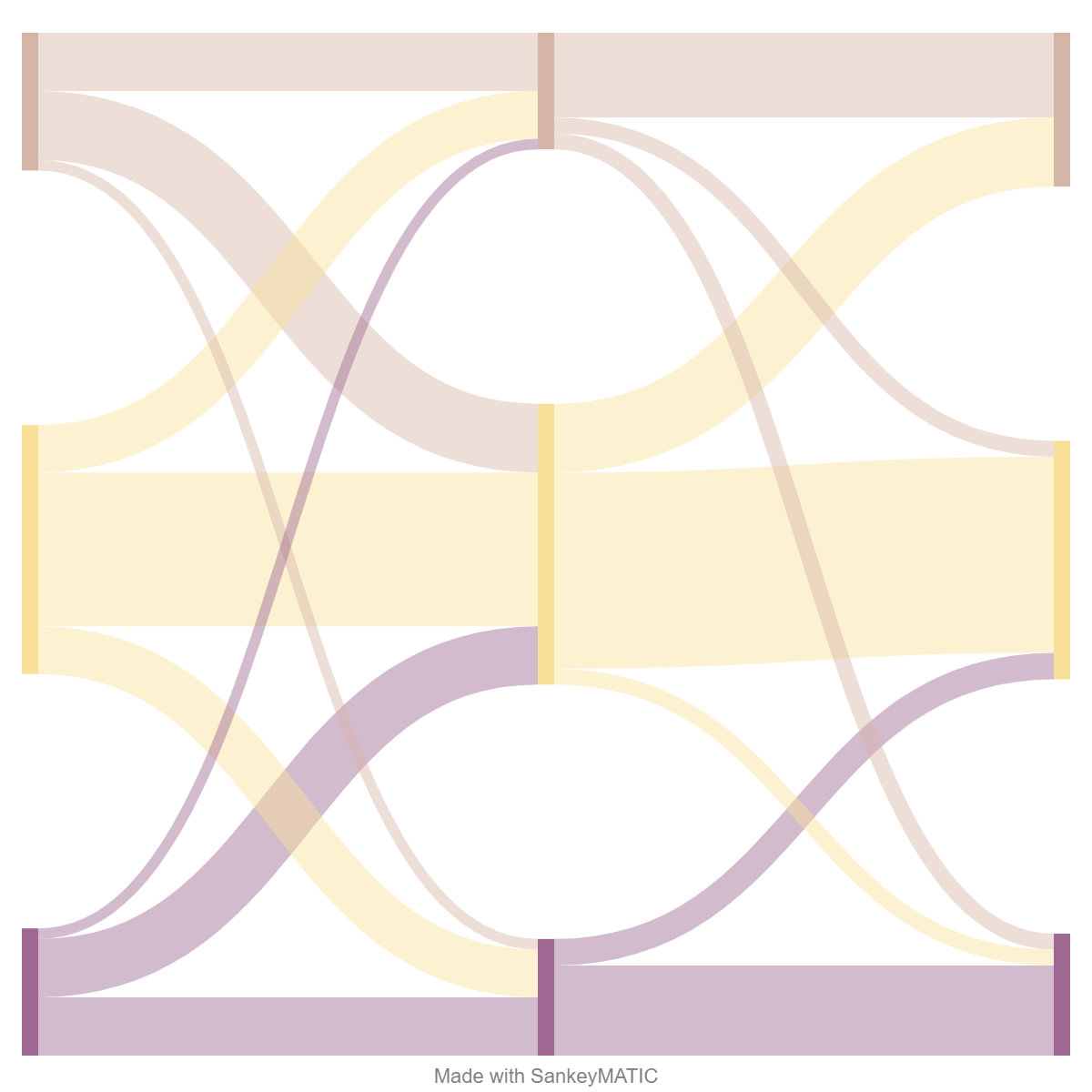


Sedentary physical activity group

Moderate-to-vigorous physical activity group

Light physical activity group

29.9%

48.5%

22.7%

23.7%

22.7%

24.7%

46.4%

54.6%

26.8%

**Fig. 1.** Sankey diagram shows the changes in physical activity level between baseline, week 12 and week 24

**Note:** Group defined according to raw scores from the self-reported Rapid Assessment of Physical Activity instrument; Please note that this analysis only included study participants who reported their physical activity level at all three time points.

**Appendix B.** Association between self-reported physical activity levels (measured by the Rapid Assessment of Physical Activity (RAPA) questionnaire) and pain score (Visual Analogue Scale /10) across the study duration.

|  | **RAPA score**  **(Spearman’s Rho)** | **P** |
| --- | --- | --- |
| **Pain score at baseline** (n=151) | -0.13 | 0.10 |
| **Pain score at week 12** (n=80) | 0.10 | 0.36 |
| **Pain score at week 24** (n=60) | -0.09 | 0.50 |
|  | **Changes in RAPA score** **(Spearman’s Rho)** | **P** |
| **Changes in pain score**  (Week 12- Baseline; n=79) | -0.06 | 0.63 |
| **Changes in pain score**  (Week 24 - Week 12; n=49) | 0.03 | 0.83 |

**Appendix C.** Association between self-reported physical activity levels (measured by the Rapid Assessment of Physical Activity (RAPA) questionnaire) and quality of life (measured by EQ-5D-5L) across the study duration.

|  | **RAPA score**  **(Spearman’s Rho)** | **P** |
| --- | --- | --- |
| **EQ-5D-5L score at baseline** (n=146) | 0.41 | 0.000 |
| **EQ-5D-5L score at week 12** (n=108) | 0.36 | 0.0001 |
| **EQ-5D-5L score at week 24** (n=118) | 0.49 | 0.0000 |
|  | **Changes in RAPA score** **(Spearman’s Rho)** | **P** |
| **Changes in EQ-5D-5L score** (Week 12- Baseline; n=103) | 0.10 | 0.32 |
| **Changes in EQ-5D-5L score** (Week 24 - Week 12; n=95) | 0.11 | 0.31 |
